# Evaluation of the effect of topical and systemic ozone application in periodontitis: an experimental study in rats*

**DOI:** 10.1590/1678-7757-2019-0140

**Published:** 2019-11-15

**Authors:** Ebru Saglam, Suzan Bayer Alinca, Tugba Zengin Celik, Uguray Payam Hacisalihoglu, Mehmet Ali Dogan

**Affiliations:** 1 Health Sciences University, Faculty of Dentistry, Department of Periodontology, Istanbul, Turkey.; 2 Van Public Oral Health Center, Van, Turkey.; 3 Bezmialem Vakıf University, Faculty of Dentistry, Department of Periodontology, Istanbul, Turkey.; 4 Yeni Yüzyıl University, Gaziosmanpasa Hospital, Department of Pathology, Istanbul, Turkey.; 5 Istanbul University, Department of Pathology, Istanbul, Turkey.

**Keywords:** Bone, Hypoxia, Inflammation, Rank ligand, Ozone

## Abstract

**Objective:**

The goal of the present study was to determine the effect of systemic and topical ozone application on alveolar bone loss (ABL) by evaluating the effect of Hypoxia-inducible factor −1 alpha (HIF-1-α) and receptor activator of NF-kB ligand (RANKL)-positive cells on histopathological and immunohistochemical changes in a rat periodontitis model.

**Methodology:**

Thirty male Wistar rats were divided into three groups: 1) Group C (control group); 2) Group SO (systemic ozone group) and 3) Group TO (topical ozone group). Experimental periodontitis was induced with a 3/0 silk suture placed at the mandibular left first molars of rats, and the suture was removed 14 days later. Ozone gas was injected intraperitoneally (0.7 mg/kg) in SO group. Topical ozone application protocol was performed using an ozone generator at 80% concentration (4^th^ grade) 90- degree probe for the duration of 30 s. Both ozone applications were carried out for two weeks at intervals of two days. Histomorphometric and immunohistochemical analysis were performed.

**Results:**

ABL was significantly lower in Group SO compared to Group C (p: 0.0052). HIF-1α- positive cells were significantly lower in Group TO than in Group C (p: 0.0043). RANKL-positive cells were significantly lower in Group SO and in Group TO compared to the control group (p: 0.0033, p: 0.0075, respectively).

**Conclusion:**

Both ozone applications decreased RANKL-positive cell counts, TO application decreased HIF-1-α positive cells counts, and SO application was found to be more effective in reducing ABL compared to control group.

## Introduction

Chronic periodontitis is an infectious disease of the periodontium and is characterized by the complex destruction between pathogenic microorganisms and the host response.^1^ Microorganisms play a role in the etiology and pathogenesis of periodontitis as well as their toxins, enzymes and similar products, by activating natural and acquired host defense through host-based enzymes. The host response protects the periodontium from local microbial attack and prevents the pathogenic microorganisms from spreading in the tissue, but it can damage periodontium cells and connective tissue, causing destruction of periodontal ligament, alveolar bone and cement.^2^

Hypoxia, a sign of chronic inflammation, is the insufficient oxygen supply to cells and tissues.^3^ The tissue oxygen requirement is increased due to metabolic activation of inflamed tissue and infiltration of the inflammatory cells in chronic inflammation. Furthermore, vasoconstriction and microthrombosis cause tissue perfusion to weaken it and a decrease in oxygen supplementation. This results in low-oxygen environment and the accumulation of hypoxia-inducible factor alpha in the inflamed area.^4^ Hypoxia-inducible factor −1 alpha (HIF-1-α) is one of the HIF-alfa subunits that are the main sensors of hypoxia.^5^ HIF-1-α is the main regulatory protein that provides adaptation to hypoxic conditions.^6^ HIF-1-α has been shown to be activated by proinflammatory signals in periodontal cells.^7^^,^^8^

It has been reported that hypoxia may play an important role in the progression of periodontal disease and the destruction of periodontal tissue.^9^ Yu, et al.^10^ (2012) stated that the lack of oxygen in periodontal tissue may accelerate the development of periodontitis. It is also known that hypoxia increases the release of receptor activator of NF-kB ligand (RANKL).^10^ RANKL is a tumor necrosis factor ligand superfamily member. It is responsible for bone resorption by stimulating osteoclastic differentiation.^11^ The binding of RANKL to the RANK receptor on the pre-osteoclast surface induces bone resorption by stimulating mature osteoclastic differentiation.^11^

Ozone is a naturally occurring compound containing three oxygen atoms. Ozone has various effects, such as antimicrobial, anti-hypoxic, immune-modulator, biosynthetic and analgesic. It has been medically used in both gaseous and aqueous forms and can be dissolved in either water or oil.^12^ Ozone allows oxygen to move inside the tissues more easily by increasing the amount of 2,3-diphosphoglycerate in erythrocytes. Ozone increases the release of nitric oxide, leading to vasodilatation and increase blood flow in tissue. An increase in free oxygen radicals leads to a change in antioxidant enzyme levels and immune system activity. It induces the production of interferon, interleukin, tumor necrotising factor and growth factors in leukocyte and endothelial cells. As a result, ozone therapy can be used for treatment in physio-pathological conditions in which the inflammatory process is intense and the immune system is triggered.^13^

There are several studies evaluating the application of local ozone in chronic periodontitis and aggressive periodontitis.^14^^–^^17^ However, to the extent of our knowledge, no clinical and experimental studies have compared the effect of systemic and topical ozone application on chronic periodontitis in periodontal tissues. The main purpose of this study is to evaluate and compare the effect of topical and systemic ozone application on the destructed periodontal tissues by means of alveolar bone loss (ABL), HIF-1-α and RANKL expressions.

## Methodology

### Study groups and experimental design

This study was approved by the Local Ethics Committee in Animal Experiments Protocol (2017/122). The sample size was calculated to provide 80% power (1-β) with a 95% confidence interval (α = 0.05); ten animals *per* group were required.^18^

Thirty male Wistar rats (*Rattus norvegicus albinus*; initial body weights ranging between 320-350 g) were dived into three groups as follows; 1) Group C (control group, n:10 rats); 2) Group SO (systemic ozone group, n:10 rats) and 3) Group TO (topical ozone group, n:10 rats). The rats were housed under standard laboratory conditions in accordance with the National Institute of Health throughout the study.^19^ The rats were housed in a room with a 12-hour light/dark cycle, temperature of 22±2° C and humidity of 45±15 %. The animals were fed with selected solid diet and water *ad libitum*.

### Experimental Periodontitis and Systemic and Topical Ozone Application

The experimental periodontitis model described in a previous study was used.^20^ General anesthesia was performed intraperitoneally with 10 mg/kg xylazine (Rompum, Bayer, Istanbul, Turkey) and 80 mg/kg ketamine (Ketalar; Pfizer, Istanbul, Turkey). The animals were placed on the operating table, allowing the rats’ mouths to be kept open and allowing access to the posterior teeth of the mandibles. Sterile 3-0 silk sutures were gently placed by a single operator (SBA) in a subgingival position around the cervixes of the mandibular left first molar and tied on the mesial side in each animal. The presence of ligatures was checked periodically. Fourteen days later, sutures were removed. Topical and systemic ozone administiration started on the same day (the day the ligatures were removed was accepted as day 0).

The ozone gas was achieved from a Longevity Ozone Generator EXT50 (Longevity Resources Inc., Sidney, British Columbia, Canada), and was injected 0.7 mg/kg intraperitoneally, according to the previous study.^21^ The topical ozone application was performed with an ozone generator (OzoneDTA generator, APOZA, Taiwan) at 80% concentration (4^th^ grade) 90-degree probe for 30 seconds.^22^ Both systemic and topical ozone applications were carried out for two weeks at two days intervals (on days 0, 2, 4, 6, 8, 10, 12 and 14).

Two days after the last ozone application, the rats were sacrificed by anesthesia overdose (30 mg/kg xylazine and 240 mg/kg ketamine).^23^ The rats’ left hemimandibles were removed and placed in 10% buffered formalin for subsequent histological processing.

### Histopathological and histometric analyses

The tissue samples were fixed in 10% formalin for 24 hours, then decalcified in 10% formic acid and embedded into parafin blocks. Histological sections (2- 4 μm) were prepared buccolingually using a microtome (Leica RM2125RT; Leica Instruments, Nubloch, Germany). The sections were deparaffinised, rehydrated and stained with hematoxylin-and-eosin (H&E) for histopathological and histometric analyses.

**Figure 1:**
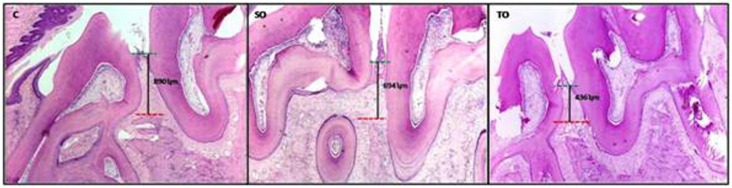
Alveolar bone loss measurement in histological images of each experimental group, (c) control group, (so) systemic ozone group and (to) topical ozone group, heamatoxylin& eosin, (40x), ʮm:micrometer red line (-----): alveolar bone crest, green line (-----): cemento-enamel junction

The static microimages of the H&E slides, captured at 40x and 100x magnifications were photographed via a microscope (Nikon Eclipse Ni-U microscope, Nikon Corporation, Tokyo, Japan) attached to a digital camera system (Nicon Digital Sight DS-L3, Nikon Corporation, Tokyo, Japan). The ABL was analyzed histometrically by measuring the distance between the alveolar bone crest and the cementoenamel juncton between the first and second molar. The mean value of the measurements of ten serial sections from each animal was calculated as bone loss (Figure 1).^24^

### Immunohistochemical analyses

**Figure 2:**
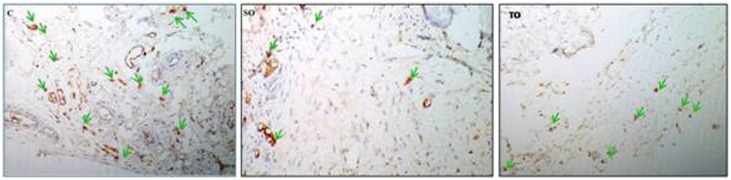
Arrows: Cells displaying positive immunoreaction with HIF-1-α immunohistochemical antibody in C, SO and TO groups, respectively (HIF-1-α antibody, 200X). (C) Control Group, (SO) Systemic Ozone Group and (TO) Topical Ozone Group, HIF-1-α; Hypoxia-inducible factor −1 alpha

**Figure 3:**
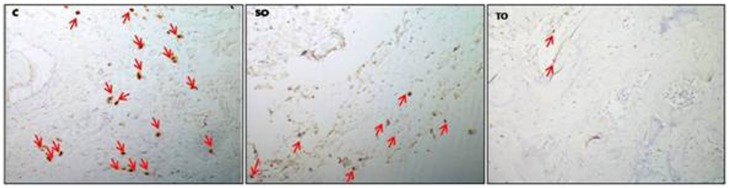
Arrows: Cells displaying positive immunoreaction with RANKL immunohistochemical antibody in C, SO and TO groups, respectively (RANKL antibody, 200X). (C) Control Group, (SO) Systemic Ozone Group and (TO) Topical Ozone Group, RANKL; receptor activator of NF-kB ligand

The paraffin-embedded sections were immunostained with CD254 (RANK Ligand) (12A668, ThermoFisher Scientific, USA) and HIF-1-α (EP118, Epitomics, USA) monoclonal antibodies as follows. The sections were firstly deparaffinized. Then, the rehydration was provided by using methanol. The sections were incubated with 6% hydrogen peroxide to block endogenous peroxidase. For the antigen retrieval, the sections were immersed in 10 mM sodium citrate buffer including 0.05% Tween 20 at pH 7.2. Then they were incubated at room temperature, using 1% bovine serum albumin for 30 minutes. The sections were incubated with anti-CD254 or anti-HIF-1-α at a concentration of 5 mg/mL and were washed three times in phosphate-buffered saline. For the localization of the antigen in the tissue specimens, a bond refine Dab detection kit containing secondary antibody (Leica Biosystems, USA), streptavidin enzyme conjugate and an AEC substrate chromogen mixture were used. The washing and the incubating time were performed according to the manufacturer instructions. H&E were used as counterstains to accentuate the visualisation of the main stain. Nonimmune rabbit serum was used as a negative control. HIF-1-α and RANKL positive cells were counted in three areas (3,79×3=11,37mm^2^) with a microscope at 200x magnification (Figure 2 and 3). The number of immunolabelled cells was detected per unit area (number of cells/mm^2^).

### Examiner Calibration

All analyses were performed by the same examiner (UPH) who was blind to the treatments. The second measurement was performed seven days after the first measurement to estimate the intraexaminer error.^23^ The student t-test was used to determine the examiner calibration in the analyses of the immunolabeling. Calibration was accepted if the difference between two measurements were not statistically significant (p>0.05).

### Statistical Analyses

Statistical analysis was performed using a software program (SPSS, version 20.0; IBM, Chicago, IL, USA). The Kolmogorov-Smirnov test was used for distributional adequacy. The significance of differences between groups was determined by Kruskal-Wallis tests, followed by Dunn multiple comparison post hoc test. The significance level was set at 5%.

## Results

**Figure 4:**
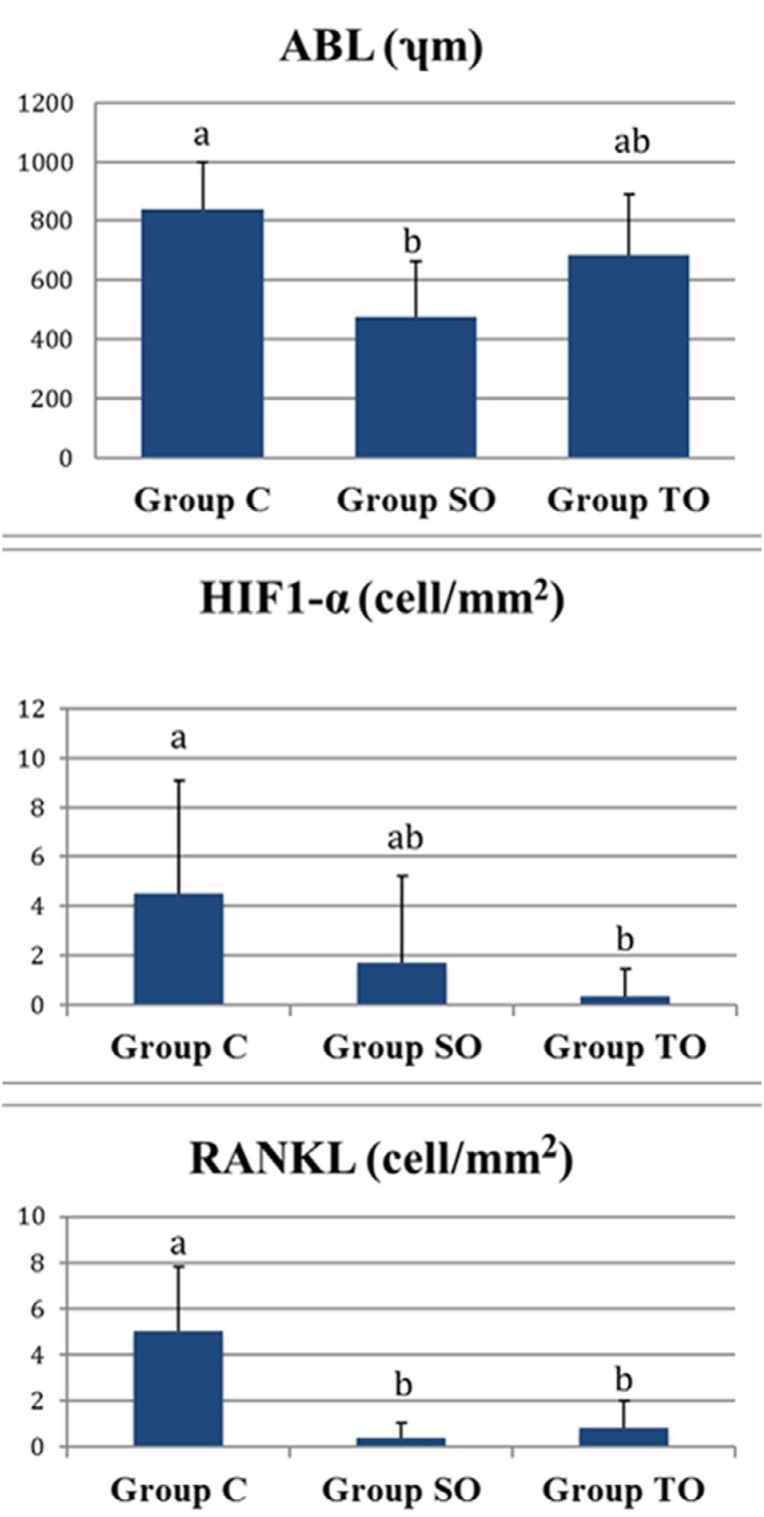
The levels of ABL, HIF-1-α and RANKL (C) Control Group, (SO) Group Systemic Ozone and (TO) Group Topical Ozone values are presented mean ± standart deviation, (p<0.05); Statistically significant difference, a, b values are statistically different. Same letters within the same row indicate no significant

A total of 30 Winstar rats (ten rats in each group) were evaluated. ABL, HIF-1-α and RANKL- positive cell values are summarised in Table 1 and Figure 4.

**Table 1:** ABL, HIF-1-α and RANKL parameters for all groups

	Control	Group SO	Group TO	
	Mean± SD	Mean± SD	Mean± SD	p1
	(SE)	(SE)	(SE)	
ABL (ʮm)	839.9± 160.3	477.3± 186.8^a^	684.7 ± 206	<0.001
	(25.70)	(29.08)	(34.66)	
HIF-1-α (cell/mm^2^)	4.53±4.36	1.73±1.54	0.37±1.11^a^	<0.001
	(0.55)	(0.81)	(0.37)	
RANKL (cell/mm^2^)	4.99 ± 2.83	0.50 ±0.45^a^	0.89±0.82^a^	<0.001
	(0.49)	(0.10)	(0.20)	

Values are expressed as Mean±SD and SE. SD: Standard Deviation, SE: Standard Error, The differences in immünohistochemical analysis results between the study groups (Group SO; systemic ozone group, Group TO; topical ozone group) were tested by Kruskal-Wallis tests, followed by Dunn multiple comparison *post-hoc* test. In each row, letter (a): Statistically significantly different from control group; p<0,05. p1: Kruskal-Wallis Test.

The highest ABL was in the control group. ABL was statistically and significantly lower in the SO group than in the control group (p: 0.0052). In the TO group ABL was lower than in the control group but this difference was not statistically significant. No statistically significant difference was observed between SO and TO groups in terms of ABL.

HIF-1-α positive cells were less in both the SO group and TO ozone group than in the control group. However, this difference was statistically significant in the TO group compared to the control group (p: 0.0043). No statistical difference was observed between SO and TO groups.

RANKL-positive cells were significantly lower in SO and TO groups compared to control group (p: 0.0033; p: 0.0075, respectively). There was no statistically significant difference in the number of RANKL-positive cells between SO and TO groups.

## Discussion

Periodontitis is a multi-factorial chronic inflammation that is caused by pathogenic bacteria and host response. In this current study, the effects of ozone application on ABL, hypoxia and RANKL levels with ligature-induced periodontitis were investigated.

Unlike chronic periodontitis, tissue destruction in a ligature induced periodontitis model tracks an acute process.^25^ Toker, et al.^26^ (2009) reported that the alveolar bone loss peaked at 11 days of periodontitis, and the ligature induced bone loss increased from day 1 to day 15. In addition, the authors stated that following the ligature placement, the process of creating experimental periodontitis with ligature should not exceed 15 days, as there may be occlusal, buccal and distal migration of the teeth. Otherwise, the severity of the destruction on tissues can significantly reduce.^27^ In this study, periodontitis was successfully induced by placing silk suture. Suture was removed on day 14 for the reasons outlined above, and ozone application started during the same session. With the removal of the suture, the cause of periodontitis was eliminated. The periodontium initiated a natural repair process. Considering these situations, the ozone application groups were compared with the control group.

Ozone therapy improves inflamed tissue oxygenation and reduces total inflammatory process. In that way, it positively affects some infectious disease outcomes. Ozone therapy can be applied via mixing a range of gases and liquids, and injecting these solutions into the body, including the vagina, rectum, muscle, beneath the skin or by autohemotherapy.^13^^,^^28^ There are some studies evaluating the effect of ozone therapys, anti-hypoxic and anti-inflammatory feature, in periodontal tissues.^14^^,^^17^^,^^29^

Although there are several studies indicating that topical ozone application with nonsurgical periodontal treatment has no additional benefit in clinical periodontal parameters,^14^^,^^30^^–^^32^ few studies have reported significant improvement in periodontal parameters in patients with periodontitis treated with scaling and root planing along with ozone application.^15^^,^^16^ Erdemci, et al.^21^ (2014) evaluated the effect of SO and TO application after tooth extraction and measured mineralized and trabecular bone and osteoid and osteoblast surfaces for determining alveolar bone healing. They concluded that postoperative long-term SO application can accelerate alveolar bone healing following extraction. In a study evaluating bone healing in the rat calvarial defect model, it has been reported that TO application increases bone formation, and is even more effective than low-level laser therapy on bone healing.^33^ It is known that ozone application is effective in the treatment of bisphosphonate-related osteonecrosis of the jaw.^34^ Ozdemir, et al.^35^ (2013) reported that TO application increased bone formation on autogenous bone grafts in the rat calvarial defect model. In accordance with these studies, in the present study, ABL was decreased in both ozone groups but this change was statistically significant only in the SO group (p:0.005).

Periodontal inflammation is thought to exacerbate local tissue hypoxia in periodontal pockets. In response to the hypoxic condition induced by the inflammatory disease, activation of HIF-1-α is increased and mechanisms that correct the hypoxic state are developed.^3^ Researches evaluating HIF-1-α levels in healthy and inflamed periodontal tissues have reported that HIF-1-α levels were higher in inflamed periodontal tissues^6^^,^^36^^,^^37^ It has been reported that hypoxic responses of HIF-1-α in periodontal tissues may produce negative feedback mechanisms in the inflammation.^6^ These finding demonstrate the importance of the level of HIF-1-α in infectious periodontal tissues. To the best of our knowledge, there is no study evaluating the effect of ozone application on HIF-1-α levels in periodontal inflammation in the literature. However, in an experimental study with soft tissue trauma, there was no statistically significant difference in HIF levels in SO application on soft tissue healing compared to the control group.^38^ In another study investigating the effect of SO therapy in diabetic nephropathy rats, HIF-1-α gene expression levels was reported to be decreased in the ozone therapy group.^39^ In the present study, HIF-1-α values were lower in both ozone application groups than in the control group. There was no statistically significant difference between SO and TO groups. However, a statistically significant decrease was observed in the TO group compared to the control group. The reason HIF-1-α was lower in the TO group may be because local application reached a more effective concentration than systemic application in the inflammation area.

RANKL leads to osteoclastic differentiation by binding to RANK.^40^ RANKL-mediated osteoclastogenesis plays a crucial role in inflammatory bone resorption. It has been reported that RANKL levels increase in the case of hypoxia in human periodontal ligament cells.^10^ In this study, RANKL-positive cells were significantly lower in both ozone application groups compared to control group. There was no statistically significant difference in the number of RANKL-positive cells between SO and TO groups. This can explain how the ozone applications reduce the number of RANKL-positive cells by decreasing the hypoxia.

Since the present study is an experimental periodontitis model, the major limitation is that it does not directly adapt to human chronic periodontitis in terms of method, dose and findings.

## Conclusion

Within the limitations of this study, it can be concluded that both ozone applications decreased RANKL-positive cell counts, TO application decreased HIF-1-α positive cell counts and SO application was found to be more effective in reducing of ABL compared to the control group. Both applications may thought to be equally effective on experimental periodontitis. Further clinical studies evaluating the adjunctive use of different ozone application procedures with periodontal therapy are needed to specify clinical benefits.
